# The patient participation puzzle: Organizing patient participation in health care networks

**DOI:** 10.1097/HMR.0000000000000498

**Published:** 2026-07-20

**Authors:** Roos G.F.M. van der Ven, Robin Peeters, Aggie T.G. Paulus, Lisann de Ruijsscher, Felice N. van Erning, Ignace H.J.T. de Hingh, Daan D. Westra

**Affiliations:** Roos G.F.M. van der Ven, PhD, Robin Peeters, PhD, Aggie T.G. Paulus, PhD, Lisann de Ruijsscher, Daan D. Westra, PhD, Department of Health Services Research, Faculty of Health, Care and Public Health Research Institute (CAPHRI), Faculty of Health, Medicine and Life Sciences (FHML), Maastricht University, Maastricht, The Netherlands; Roos G.F.M. van der Ven, PhD, Ignace H.J.T. de Hingh, PhD, Department of Oncology and Developmental Biology (GROW), Faculty of Health, Medicine and Life Sciences (FHML), Maastricht University, Maastricht, The Netherlands; Roos G.F.M. van der Ven, PhD, Felice N. van Erning, PhD, Ignace H.J.T. de Hingh, PhD, Department of Research and Development, Netherlands Comprehensive Cancer Organisation (IKNL), Utrecht, The Netherlands; Aggie T.G. Paulus, PhD, School of Health Professions Education (SHE), Faculty of Health, Medicine and Life Sciences (FHML), Maastricht University, Maastricht, The Netherlands; Felice N. van Erning, PhD, Ignace H.J.T. de Hingh, PhD, Department of Surgery, Catharina Hospital, Eindhoven, The Netherlands

**Keywords:** alliances, health care networks, interorganizational, patient engagement, patient involvement, patient participation, patient partnership

## Abstract

**Introduction:**

Health care is increasingly organized in interorganizational networks, with patient participation gaining prominence to better tailor care to patient needs. However, these often occur independently, limiting understanding of how to organize interorganizational patient participation. Poorly implemented participation risks draining time and energy from vulnerable groups without creating meaningful change.

**Methods:**

We conducted an in-depth case study of a multihospital network, using 23 semistructured interviews with patients, health care staff, and network administrators, 77 hours of observations, and 276 documents to explore how interorganizational patient participation functions.

**Results:**

Our findings reveal the *patient participation puzzle*: the tension between the aspiration for patient participation at all interorganizational levels and its potential burden and unclear impact. Four interrelated themes emerged: aim, organizational structure, participant attributes, and impact. We distinguished participation within the network’s operational level, closely tied to care delivery, and in network strategy, where patients’ contributions are less clearly defined.

**Conclusion:**

Patient participation in network strategy often lacks clear aims, leaving patients’ roles unclear and imposing undue burden. Key participant attributes like experiences and perspectives influence engagement but can also pose challenges. Participation’s impact is not inherent; without clear aims, structures, and roles, it risks becoming symbolic and burdensome without meaningful impact.

**Practice Implications:**

Organizing patient participation in health care networks is quite the puzzle. To avoid window dressing, network managers should acknowledge patient burden, set clear goals, act on patient input, and organize carefully to prevent tokenism. Accreditation bodies and policymakers should prioritize guidance and support over mandates to ensure meaningful impact.

Health care systems increasingly face complex challenges, including the constant need to improve quality, reduce costs, enhance population health, promote health equity, and improve provider experience (Nowell & Kenis, 2019; Nundy et al., 2022). Two developments have emerged in response: increasing collaboration between health care organizations (Raab & Kenis, 2009) and growing patient involvement in decision-making (Bombard et al., 2018). Interorganizational networks have become a dominant model for organizing collaborative care efforts (Nowell & Milward, 2022). They are defined as structured collaborations between multiple independent organizations that coordinate resources and activities to achieve shared goals, also known as coalitions, collaboratives, (multistakeholder) alliances, consortiums, and partnerships (Nowell & Milward, 2022). In parallel, health care has moved toward more active patient involvement, especially at the individual level (e.g., shared decision-making), and to a lesser extent at the organizational level (e.g., service design and policy development) (Karam et al., 2018). However, patient participation in networks remains in its early stages, with limited understanding of how to meaningfully engage patients at the interorganizational level, despite growing political momentum for participation at all system levels (Epstein et al., 2010), and its inclusion in network accreditation standards (Wind et al., 2021). This lack of clarity is concerning, as literature shows that patient involvement does not always lead to meaningful impact, raising ethical and practical concerns about asking vulnerable groups to invest without clear benefits (Van De Bovenkamp & Trappenburg, 2009). As health care networks become more prevalent, identifying effective strategies for meaningful patient participation is increasingly urgent.

In recent decades, patient participation has gained attention in both research and practice (Bombard et al., 2018). A key distinction exists between individual participation, focused on one’s personal health, and collective participation, which concerns the health of the collective (Malfait et al., 2018). Collective participation, in which patients are involved to shape health care services and policies for the collective good, is thought to improve care quality, clinical outcomes, and patient experiences (Castro et al., 2016; Westerink et al., 2023). Research on collective participation has primarily focused on organizational patient participation, suggesting that involving patients can strengthen health care governance (Bombard et al., 2018; Malfait et al., 2018). However, organizational patient participation is not inherently meaningful and may become tokenistic if poorly designed (Bombard et al., 2018). Its role in interorganizational networks has received limited attention. While several frameworks exist on patient participation in research networks (Gonzalez et al., 2023; Greenhalgh et al., 2019), participation in service delivery networks remains underexplored (Tremblay et al., 2021). These contexts warrant separate consideration given their differing goals and dynamics (Modigh et al., 2021). As a result, little is known about how to implement meaningful patient participation in health care networks, despite evidence that engaging community stakeholders, such as patients, can enhance network effectiveness (Tremblay et al., 2021; Provan & Milward, 2001).

This study aims to better understand interorganizational patient participation by addressing the research question: *How does interorganizational patient participation work?* We build on the existing concept of collective patient participation, defined as the involvement of patients in governance and decision-making processes (Castro et al., 2016), and extend it by examining how this is enacted within an interorganizational network spanning multiple organizations. In doing so, this study bridges two areas that are rarely integrated: organizational-level patient participation and interorganizational network governance, making their interplay explicit. Through an in-depth case study, it explores patients’ and professionals’ experiences, challenges, and expectations, highlighting factors that shape meaningful participation. It addresses gaps in understanding patient participation at the interorganizational level, contributing to the limited knowledge and laying a foundation for future research. By empirically investigating a network that implemented two forms of participation, the study offers valuable insights into interorganizational participation at both the operational and strategic network levels. These findings represent a first step in translating the concept of patient participation into the context of health care networks, supporting these networks in organizing meaningful patient involvement and informing policy and accreditation processes aimed at embedding patient voices more effectively.

## Theory

Over the past decade, patient participation has become a self-evident health care practice (Bombard et al., 2018). Patient participation refers to patients’ involvement in shaping the assessment, design, organization, and delivery of health care services, drawing on their experiences and preferences (Tritter, 2009). It spans activities from individual care decisions to service development and policy-making (Castro et al., 2016; Malfait et al., 2018). Patient participation has become a central principle in contemporary health care systems (The National Health Service NHS Commissioning Board, 2015). For example, this commitment is reflected in several key declarations within the European Union (Active Citizenship Network, 2002). Policy frameworks, such as World Health Organization (WHO) Europe Framework for People-centred Health Systems, emphasize that embedding patient and family engagement throughout service design and delivery is essential for achieving truly people-centered and integrated care (World Health Organization WHO Regional Office for Europe, 2016). Several frameworks emphasize participation in areas such as treatment decisions, service evaluation, education, and research (Castro et al., 2016; Tritter, 2009). This involvement can be understood as a continuum, ranging from nonparticipation or mere consultation to full partnership (Arnstein, 1969; Carman et al., 2013). However, while this continuum suggests a linear progression, participation is not linear but rather dynamic, context-dependent, and shaped by iterative processes (Tritter & McCallum, 2006). Participation thus can take multiple forms, but it also takes place across multiple levels: direct care, organizational quality improvement, and system-level policy-making (Carman et al., 2013), and reflects the broad scope of patient participation across different domains of health care, which are often broadly classified into individual and collective participation (Castro et al., 2016).

### Individual versus collective patient participation

Individual patient participation refers to patients influencing their own care through dialogue that integrates their preferences, abilities, and experiences with professional expertise. A key example is shared decision-making, where professionals and patients exchange information, discuss options, and jointly make or defer decisions (Stiggelbout et al., 2015).

Collective patient participation involves patients or their representatives contributing experiential knowledge, alongside professional expertise, to shape health care services and policies for the collective good (Castro et al., 2016). Extending beyond the individual provider–patient relationship, it includes organizational (e.g., patient advisory councils) and interorganizational (e.g, patient panels providing input on strategic decisions of networks spanning multiple organizations) involvement (Bombard et al., 2018). While interorganizational participation remains underexplored in scientific literature, there is some evidence on organizational participation. This has been linked to greater effectiveness and efficiency (Simpson & House, 2002), and typically involves activities such as guideline and policy development, and care delivery improvement (Malfait et al., 2018). Organizational patient participation strategies can be positioned along a continuum of engagement, where higher-engagement approaches, such as co-creation and partnerships, tend to foster more meaningful improvements, while lower engagement strategies yield limited outcomes and risk being perceived as tokenistic (Bombard et al., 2018). Moreover, participation often fails to make an impact when there is a mismatch in expectations or methods (Ocloo & Matthews, 2016; Tritter & McCallum, 2006). Meaningful involvement requires clear objectives and recognition of the different forms participation can take (Tritter & McCallum, 2006). Insights from this organizational literature may be relevant for interorganizational contexts, but this connection has not yet been examined.

### The interorganizational setting

Care across organizations is often organized within interorganizational networks, broadly distinguished into serendipitous networks and purpose-oriented networks (Nowell & Kenis, 2019). Serendipitous networks emerge unintentionally through loosely connected actors contributing to a shared outcome, such as referral or disaster response networks (Nowell & Milward, 2022). In contrast, purpose-oriented networks, also known as multistakeholder alliances or partnerships, involve three or more autonomous organizations intentionally collaborating toward a common purpose (Nowell & Milward, 2022). They have bounded membership, governance, and collective action towards major health care challenges (Nowell & Kenis, 2019; Nowell & Milward, 2022). Importantly, these networks are not health systems but collaborations of autonomous hospitals without shared ownership. Such purpose-oriented networks differ from affiliations in that they are formed deliberately to achieve specific goals. Such networks are increasingly common and promoted by governments and patient advocacy groups (Nowell & Kenis, 2019).

Purpose-oriented networks often arise as a response to wicked problems (Ferlie et al., 2013). Health care systems are inherently complex and face challenges that cannot be solved by a single organization, requiring coordination among the participating organizations (Ferlie et al., 2013; Nowell & Kenis, 2019), making it crucial that the right stakeholders are at the decision-making table (Peeters et al., 2023b). In such networks, engaging the served community is associated with greater network effectiveness (Provan & Milward, 2001), making their inclusion in the network’s decision-making intuitively logical. While patient participation in interorganizational networks is gaining attention, its practical implementation remains underexplored. One study showed it can help ensure patients’ perspectives on the benefits and drawbacks of practices are considered, but also highlighted the complexity of balancing interdependence with organizational autonomy (Tremblay et al., 2021). Our study explores how patient participation unfolds in purpose-oriented health care networks.

## Methods

This study employed an in-depth qualitative case study to explore interorganizational patient participation. Case studies are particularly well suited for examining emerging phenomenon with limited prior research, such as interorganizational patient participation (Savin-Baden & Major, 2013; Yin, 2018). The study adhered to COREQ guidelines (Tong et al., 2007), and ethical approval was obtained from Maastricht University (FHML-REC/2022/047).

### Setting and case

This study was conducted in Dutch oncological care, where regional networks have existed for several years (Integraal Kankercentrum Nederland IKNL, Citrien, Nederlandse Federatie Kankerpatienten NFK, & SONCOS Federatie Medisch Specialisten, 2022). Cancer is the leading cause of death in the Netherlands, responsible for 28% of mortalities in 2023 (Centraal Bureau voor de Statistiek CBS, 2024). Since 2012, mandated minimum treatment volumes for certain cancers have increased regionalization and led to multihospital networks (Foundation For Oncological Cooperation SONCOS, 2021; Organisation for Economic Co-operation and Development, 2023). This study focuses on a network in southeast Netherlands, comprising nine hospitals, and one radiotherapeutic center. Serving approximately 10% of the Dutch population, it aims to provide optimal cancer care region-wide. Initial network discussions began in 2013, with first quality-improvement meetings in 2015 and a binding collaboration agreement in 2018.

The network comprises multiple organizational layers (Supplementary file 1, Supplemental Digital Content 1, http://links.lww.com/HCMR/A201) and operates as a shared, participant-governed network in which every organization interacts with every other organization to govern the network (Provan & Kenis, 2007). Although organizations vary in size and resources, power distribution within the network is relatively balanced, consistent with a participant-governed model (Provan & Kenis, 2007). At the highest level, the executive committee, made up of directors from all affiliated organizations, handles budgeting and strategic alignment across institutions, advised by the Regional Oncology Committee. This group, composed of each center’s internal oncology committee chairs, often a medical specialist and a manager, meets quarterly to address overarching strategic network issues. At the network’s operational core are 15 service delivery working groups, most pathology specific (eg, breast cancer) and some overarching (eg, supportive care). They include one to three representatives per organization, mainly medical specialists, and meet quarterly to discuss regional care pathways, share knowledge, and exchange best practices. Three network-employed administrators connect the strategic and operational levels, attending all meetings.

The network integrates patient participation at both the operational and strategic levels. At the operational level, four out of 15 working groups included patient representatives for several years, three from the national patient organization and one from the network, participating in meetings where health care professionals discussed care pathways, standardization, and best practices. In the other eleven groups, patient participation was discussed but not implemented based on the argument that one patient could not sufficiently represent the diversity of the network’s patient population. At the strategic level, the network established a Patient Advisory Board. In 2023, the network applied for an accreditation program, evaluating comprehensive cancer networks based on Europe-wide standards (Wind et al., 2021), which was granted in 2024. This program recommended integrating patient participation throughout all network levels, which led to the establishment of the Patient Advisory Board. By 2025, eight of ten member organizations were represented. The board also included one of the network administrators, a medical specialist (who also joins the Regional Oncology Committee), and a quality advisor. This case provides a rich setting to study patient participation at both operational and strategic levels, revealing how it unfolds across the network.

### Data collection

We used semistructured interviews as the primary data source, with documents and field notes used for triangulation to confirm and contextualize themes emerging from the interview data (Table 1) (Savin-Baden & Major, 2013). Between April and June 2024, two researchers (RvdV and LdR) conducted interviews across five groups: (1) Patient Advisory Board members (PAB), (2) Regional Oncology Committee members (ROC), (3) network administrators from the network under study (NA), (4) network administrators from other Dutch regional oncology networks (NA), and (5) representatives from the national cancer patient federation (PF). Network administrators from other Dutch regional oncology networks (group 4) and representatives of the national patient federation (group 5) were intentionally approached to provide an external perspective on patient participation and to understand why they have not yet implemented it. A total of 23 one-hour interviews with 24 respondents (including one double interview) were conducted either via Microsoft Teams or face-to-face, depending on participants’ preferences. From the groups within the network (the case in this study), we invited all eligible individuals we were permitted to approach: every patient in the Patient Advisory Board (N=8), all network administrators active in this network (N=3), and for the Regional Oncology Commission (ROC), one ROC member per hospital (N=10), using purposive sampling to capture diverse backgrounds (eg, medical specialists, advisors, managers). The maximum of one interview per organization was agreed upon with the network, given the time investment required from board members in the ROC. In addition, to include an external perspective, we also invited all network administrators from other regional oncology networks in the Netherlands (N=6), as well as two representatives from the Dutch Federation of Cancer Patients, who were willing to participate in a joint interview. Reasons for nonparticipation included feeling uncomfortable with an interview (N=1, PAB), nonresponse (N=1, PAB), and a lack of time (N=2, ROC). One network did not have a network administrator in place at that time and could therefore not be approached. Data saturation was reached within certain subgroups, including the Patient Advisory Board, network administrators, and the Regional Oncology Committee, as indicated by the absence of new themes after 4–5 interviews within these groups. For smaller subgroups, saturation could not be guaranteed due to their limited size; however, this was not considered problematic, as these interviews primarily served as an additional external perspective to contextualize the findings. Two researchers (RvdV and LdR) attended a Patient Advisory Board meeting to request permission to approach members individually for interviews. We asked the network administrators in the other groups to request permission from their colleagues to be contacted. Participants received study information via email and provided informed consent. The interview guides were developed by the first and second author and reviewed by the research team. The interview guides contained core questions applicable to all groups, with a slightly stronger focus placed on aspects specific to the specific roles (Supplementary file 2, Supplemental Digital Content 1, http://links.lww.com/HCMR/A201). Interviews were audio-recorded, automatically transcribed using Amberscript, and manually reviewed.

**TABLE 1:** Collected data

Interviews	n
Patient Advisory Board (PAB)	6
Regional Oncology Committee (ROC)	8^a^
Network administrators from the network under investigation (NA)	3^b^
Network administrators from other Dutch regional oncology networks (NA)	5
Representatives from Dutch cancer patient federation (PF)	2^c^
Total	23 (24 respondents)
Documents	Documents
Network Documents and Network Accreditation Forms	8
Meeting minutes/discussion notes of:	
the Patient Advisory Board	21
the Regional Oncology Committee	22
working groups in which patient participation is integrated (n=4)	225
Total	276
Observations	Hours
Patient Advisory Board meetings	5
The network’s working groups	68
Regional Oncology Committee	4
Total	77

^a^ One Regional Oncology Committee representative also takes place in the Patient Advisory Board.

^b^ One network administrator from the network participates in the Patient Advisory Board.

^c^ Double interview.

In addition to interviews, we collected 276 pages of documents and 77 hours of observational data for triangulation. Observations covered strategic-level Patient Advisory Board meetings and operational-level meetings of the 15 working groups, with field notes taken throughout. Documents, obtained via the network’s intranet, to which the first author was granted access, included meeting minutes, discussion notes, and general network materials. Both data sources informed analysis of the design, implementation, and role distribution of patient participation, its intended goals and discussion topics, and stakeholder attitudes toward patient participation in general and its organization and development in this case.

### Analysis

We analyzed interviews inductively using an integrated thematic approach (Braun & Clarke, 2006) in Atlas.ti 24.1.1. Three researchers (RvdV, RP, and LdR) independently open-coded two random transcripts, compared results, and developed a preliminary codebook (Green & Tohorogood, 2018). Any discrepancies were addressed through discussion among the researchers until consensus was achieved. After coding six interviews from different respondent groups, the codebook was refined in discussion with the research team. The first and second author coded the remaining transcripts, with regular team discussions to refine codes and insights. Open coding was followed by iteratively refinement and axial coding to group codes and explore connections, resulting in four themes: (1) aims of interorganizational patient participation, (2) its organization, (3) participant attributes, and (4) its impact (Supplementary file 3, Supplemental Digital Content 1, http://links.lww.com/HCMR/A201). Themes were considered based on their relevance to the research question and their conceptual richness. Documents and observations were used to triangulate and contextualize the findings (Savin-Baden & Major, 2013). We then drew on network governance and patient participation literature to interpret how interorganizational structures influence patient involvement and to highlight tensions between inclusivity and efficiency that current frameworks only partly capture.

To ensure trustworthiness, we applied several strategies: reflexivity to address the diverse backgrounds of the research team and minimize bias; data triangulation through interviews complemented by document review and observations; consultation with health care management researchers outside the author team, who critically reviewed our interpretations and provided feedback to enhance rigor; and member engagement via interim presentations to network administrators, the Patient Advisory Board, and the Regional Oncology Committee. These measures strengthened credibility and transparency throughout the research process.

### Reflexivity

Our research team was highly diverse, both in disciplinary background and professional experience, ranging from students to full professors, including health care management researchers and medical specialists. This diversity enriched the analysis by bringing multiple perspectives and interpretations. At the same time, we acknowledged that these varied lenses and levels of experience could influence how data were understood. To mitigate this, we engaged in regular team discussions to challenge assumptions, documented analytic decisions, and sought consensus through iterative coding. These steps aimed to enhance transparency and minimize potential bias throughout the research process. The researchers who conducted the interviews were not part of the respondents’ organizations and had no direct power relationship with them, which helped ensure openness and reduce potential social desirability bias.

## Results

Respondents expressed diverse opinions on the aim, organization (eg, level of meaningful implementation and structure), participant attributes, and impact of interorganizational patient participation. Results highlight a distinction between operational-level (e.g., pathology-specific working groups) and strategic-level participation (e.g., Patient Advisory Board). The following sections present the results around these four themes and examine their interconnections, considering the distinction between the operational and strategic levels.

### Aim of patient participation

As one interviewee mentioned: *“If you ask me whether a patient advisory board is necessary, I can only say yes, because in a context of [a network] in which patients are actually central, saying no to patient input, that is simply not an option”* (NA5). Driven by the principle that health care networks exist to serve patients and must therefore actively involve them, respondents widely recognized interorganizational patient participation as necessary. External factors strongly shaped this view. For example, The Dutch Foundation for Oncological Cooperation (SONCOS) is frequently mentioned by both Regional Oncology Committee members and network administrators for stating that patient input is essential: *“If you read [the report of] SONCOS, it actually makes perfect sense: it clearly states that you simply need input from the groups you are working for”* (NA5), referring to the SONCOS standards report, which states that an oncology network must ensure the structural involvement of patients in the organization and development of the network as a whole (Federatie Medisch Specialisten, 2025, p. 14). Respondents also noted the influence of the accreditation process, where patient participation was a key criterion. Several documents confirm that this requirement prompted the network to establish a Patient Advisory Board at the strategic level. One accreditation standard states: *“The (…) network has established mechanisms to incorporate the voice and opinions of patients and families”* (Document 180624OECI). Although the network had long considered introducing strategic-level patient participation, the accreditation process accelerated its creation before the aim was fully defined. As one respondent explained:

*“It went particularly fast because we had the accreditation going on, and patient participation was literally one of the criteria for that. So, there was a sense of urgency, that is my impression, we had to have such an advisory board in place quickly. And now I have the feeling that (…) the entire network is actually still searching for, what are they really useful for? Where can we involve them? (…) Not much has been discussed in detail about that yet.”* (ROC1)

Respondents noted differences in the value of the two levels of interorganizational patient participation. Observational data revealed that nearly all recognized the added value at the operational level: *“A poll (…) [conducted during the meeting] showed that 82% of the attendees who responded think that having a patient join this working group is meaningful”* (Observation#2). Respondents described the main aim as to assess whether care pathways align with patients’ needs. As one respondent mentioned: “*From the experience of these patients, you can really deduce: what are we actually doing in this entire care pathway, what could still be improved, what could be changed?”* (ROC2). Only a few questioned this value, concerned that input from one or two patients might not adequately reflect the broader patient population.

There was less agreement on the aim and value of patient participation at the strategic level. As one field note describes: *“Although patient participation is unanimously desired, it is still unclear to them what it should be used for”* (Observation#6). Suggestions mentioned in interviews included articulating patient voices, advising on network matters, strengthening local patient councils, offering solicited and unsolicited advice, sharing local patient participation practices, and sharing regional perspectives, but these remained broad and inconsistent. Most respondents struggled to define a clear purpose or relevant topics for participation at the strategic level: “*I find it difficult. I do not know what their role could be (…) The question is: what is this [Patient Advisory Board] going to mean, what will they contribute?”* (NA3). Patient representatives shared this uncertainty: *“I find that a difficult question [to explain the aim], the direction and content (…) are still taking shape, and I do not yet have a clear idea of where it is heading”* (PAB4). The abstract nature of strategic-level discussions, often disconnected from direct patient experiences, made it hard to set actionable goals, as illustrated by the following observational note: *“There is still no clear idea of what the Patient Advisory Board is actually going to do. (…) Discussion about their purpose takes up much of the [PAB’s] meeting time.”* (Observation#38)

Opinions varied on who should define the aim of strategic-level patient participation, ranging from patients themselves to the Regional Oncology Committee, or both. Most respondents from all interviewed groups favored patients defining it, arguing it should not be decided for them, although some felt it should not be left entirely to the patients: “*I feel that too much responsibility is being placed on us (…). I think you are asking your patients too much. We do not know this [network].”* (PAB2). These respondents felt the network should set the goal before launching the initiative. In one organization, patients declined to join the network’s Patient Advisory Board, citing unclear purpose and limited perceived contribution at this strategic level. Some even questioned whether the board should exist without a defined aim. In other networks, this uncertainty was the reason strategic-level participation had not yet been implemented, because: *“If it is not necessary, we should not be doing this”* (NA5).

*“These people are being asked, ‘Hey, you are now the Patient Advisory Board. Go ahead and figure out your purpose, because we do not know it’. Why are they there then? Why set them up if you do not know what their purpose is? You are letting people (…) figure out their own role and goal. (…) You do not set up a committee and then say, ‘Just figure out what you can do’.”* (ROC8)

### Organization of interorganizational patient participation

Respondents noted that strategic-level patient participation was initiated without a clear plan, assuming structure will develop over time. Various forms of patient participation exist, and interviews revealed diverse views on the ideal organizational form. Some favored a large, diverse patient panel to capture a wide range of perspectives with an evaluative focus. Others preferred a small, selective group of patients working closely with professionals from the outset in a co-creation model. Another approach emphasized a Patient Advisory Board composed of patients participants from the different service delivery working groups to bridge those levels. Some suggested combining these methods. Preferences were closely linked to the intended aim of participation, yet there was little consensus on that aim and no shared vision of the ideal organizational form.

*“So I think it really depends on the tasks and responsibilities you assign to such a Patient Advisory Board. What is the goal? Why are they there? And I think, actually, if that is clear, then you can very well align what you need for that.”* (NA5).

Documents revealed the network’s attempts to implement operational-level participation since 2015, with the topic appearing in each group’s annual reports since then. In 2016, patient participation was introduced in two working groups. A patient representative joined the meetings to provide input on the network-wide care pathways and share insights from the national patient organization. While expansion to all 15 working groups was planned, by 2025 only four implemented participation. While documents did not specify why, some respondents believed concerns about interference with professionals’ work played a role. As one patient recalled: *“Initially, there was a lot of resistance from the independent specialists. (…) Let us be honest, it is often about money (…) they were afraid we would interfere with that”* (PAB1).

Respondents identified two additional challenges to interorganizational patient participation. First, maintaining engagement is difficult due to changes in representative’s health, circumstances, or even death, especially in vulnerable populations like oncology: *“We have had quite a few patients who have passed away in the meantime (…). So how do you deal with that?”* (ROC4). Second, networks are not legal entities, and so the Patient Advisory Board has no formal rights, operating voluntarily without authority.

*“What is my authority? To put it simply, in a condescending way, nothing. (…) I do believe the patient should have a say in it, but that is different from responsibility, and even different from authority. (…) Maybe ‘having a say’ is the best term for it, (…) but I do not have the authority to say, ‘This must be different’”* (PAB2).

While patient input is valued, respondents emphasized it should not be the sole deciding factor: “*We can say: ‘We will take this into account in our further decision-making’ but it should not be the case that if they say ‘no’, while (…) we believe that it would truly add value (…), that it gets blocked”* (NA5). Given lack of formal mandate, respondents emphasized that they would appreciate a greater clarity regarding the organizational framework and expectations. *“How do you make decisions? What is the role of the chairperson? How is the connection with the ROC? (…) How are the compensations arranged for these people? Those kinds of things. (…) What exactly are we? (…) you can perfectly define together what you are and what you are not.”* (LP2)

### Participant attributes

Respondents noted that no strict criteria were set for patient representatives’ skills or experiences, aiming for broad inclusion. The focus was on recruiting one representative per member organization in the Advisory Board. There is no consensus on whether every organization should be represented. Some believe it is crucial for communication with local patient advisory councils: *“I think that is important because the Patient Advisory Board needs input from the underlying organizations”* (PAB9). Others argue that patient representatives should reflect the collective patient voice: *“Primarily, I believe you are there for all ten [organizations]. It is about how the network functions as a whole. (…) I see my primary role as representing [the network] as a whole”* (PAB2). A network administrator similarly described: *“I think it really is about the representation of the patient’s voice (…). It is not about what he or she finds important, but what the group considers important, and ensuring that voice is clearly heard”* (NA4). Some questioned the feasibility of having every organization represented, suggesting a balanced distribution: *“Ideally, you would want representation from each organization, but that is probably not achievable, or not yet achievable. Then at least make sure there is a good distribution, with representation from smaller and academic hospitals, to ensure diversity is reflected”* (ROC1).

Beyond organizational representation, opinions differed on the ideal composition of patient representatives at the strategy level. Some favored a heterogeneous group to reflect the diverse population, while others preferred a carefully selected group with specific skills suited for strategy-level discussions. Key skills highlighted included relevant work experience (eg, in health care), the ability to engage in strategic or system-level discussions, persuasive communication, confidence, and the capacity to gather input from their communities. The most cited skill was the ability to think collectively, rather than from personal experience: *“You want someone to be able to look a bit overarching. (…) I think someone (…) should think a bit more overarching, a bit detached from his or her own story, because that is the principle of representing an entire group”* (ROC1). However, both professionals and patients acknowledged the difficulty of finding such volunteers: “*It is a scarce resource: patients who are willing and able”* (PAB2).

Although the study focuses on an oncology network, not every member of the Patient Advisory Board is or has been an oncology patient. One joined via their local hospital’s client council. When the network began recruiting patients for the Patient Advisory Board, it invited the client or patient councils of all member organizations to nominate representatives, which led to this participant’s involvement. *“I have absolutely no knowledge about [oncology], and fortunately, I also do not (…) have any personal experience with it myself. So, I am not an expert by experience”* (PAB1). Interviews revealed that both professionals and fellow patients on the board were unaware that a representative without direct oncology experience had joined. Most believed participants in the Advisory Board should have direct oncology experience, either as a patient, through a spouse or other close relation with cancer, or through work experience. *“You need to draw your affinity from your own experiences, such as being a patient, or having worked in that sector, or being an informal caregiver, or, I do not know, through family or something like that. But I do think it is important to be able to establish that connection to some kind of affinity”* (PAB7). The same applied to the operational level, where it was indicated that they would preferably include a patient with experience of the specific condition, as illustrated by the following observational note:

*“One patient always participated in the meetings as an expert by experience but is now stepping down. The person taking over is not an expert by experience, but a patient representative. The attendees clearly expressed their regret about this change. As a result, efforts are underway to find a new patient with experience of this condition to join the meetings.”* (Observation#27)

### Impact of patient participation

Respondents generally view patient participation as valuable for integrating the patient perspective into decision-making and avoiding policies based solely on professional assumptions. *“Would it be necessary? Yes, I definitely think so, because (…) we come up with so many things for the patient without involving them. Maybe we should start by including the patient and asking what they actually want”* (ROC1). It is especially valued for optimizing care pathways and addressing regional differences in patient needs, mainly at the operational level. Some respondents see potential at the strategic level, but struggle to identify concrete topics where patients could contribute, highlighting a gap between perceived and actual impact.

Several respondents questioned the added value of patient participation at the strategic network level. Some doubt whether its perceived value is genuinely felt: *“They say they think it is great and fantastic (…), but in practice, they just find it ‘okay’. It is all a bit of a performance”* (NA2). Others are skeptical about the Boards effectiveness, citing a lack of clear aims and tangible impact. They worry participation risks becoming tokenistic and question the fairness of involving patients in discussions where their input may not be meaningfully used.

*“I see much less value in such a Patient Advisory Board. (…). Right now, it almost feels like an obligation rather than something I truly see a lot of value in. (…) Sometimes it is all about details or things that make you wonder: do you really need to burden a patient with that? What is the added value, so to speak?”* (NA3).

For some respondents, the unclear value of patient participation raises concerns about the burden on patients, prompting questions about whether it should be organized at the strategic level, given the unclear aim and doubts about its impact. According to respondents, participation often requires a significant voluntary commitment from individuals already facing health challenges, especially in vulnerable populations. As one patient noted: “*It is already hard enough for us to find someone for [the patient council in our own organization]. (…) They say: ‘I have got my hands full!’ And I think that is especially true when it comes to cancer”* (PAB2). Some respondents criticized networks for promoting patient participation without clear goals.

*“I find it absolutely worthless to say: ‘here you are, now go figure out what you can do’. I would almost call it an insult. In a way, you might even be taking advantage of them (…). Because if you cannot even figure out yourself what role a patient advisory board should have, why do you want one? Is it just because the whole world thinks it is a good idea? (…) They need to have a purpose. You do not establish one just because everyone else does, but we do not even know what to do with it. I will just say this: that is not a role I would want to take on.”* (ROC8)

### Interrelationship of the patient participation dimensions

**FIGURE 1:**
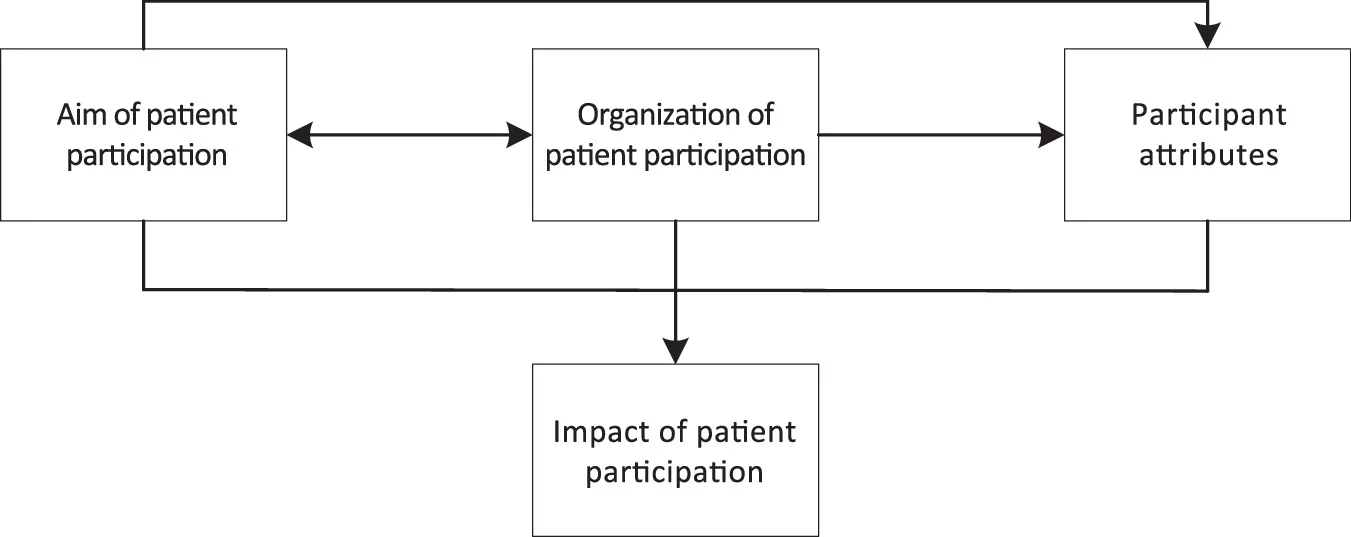
Interrelationship of the patient participation dimensions.

The dimensions of patient participation: aim, organization, membership, and impact, are interrelated, creating a dynamic system where decisions in one area affect the others (Figure 1).

The aim of patient participation is closely tied to its organization (eg, level and structure). If the aim is co-creating strategy, a Patient Advisory Board is often used, while a patient panel is preferred for feedback on predefined policies. Thus, the aim shapes the structure, as one respondent mentioned: *“First, it is about: where are we headed? You know, the why. And then you determine how we are going to get there.”* (ROC4). However, this relationship is reciprocal: networks may first establish a Patient Advisory Board and later define its aim, suggesting that the structure can influence the aim. As mentioned by a respondent: *“That has now started, such a Patient Advisory Board, and in the beginning, it takes a lot of time to shape it. Then the next step is: what will you actually involve them in?”* (ROC1). This creates a reciprocal relationship between the aim and the organization of patient participation.

The organization of patient participation also influences membership requirements. In a Patient Advisory Board, members need skills like collaboration and representation, while in a panel, personal experience is more important. The aim can indirectly shape both the organization and membership requirements. However, membership attributes do not dictate the aim or structure of patient participation.

Similarly, the aim of patient participation shapes the required membership attributes. For example, for strategy co-creation, members may need strategic knowledge and analytical skills. One patient explained: “*There are very few people who can make the leap from the n=1 experience to understanding how it works at the tactical level, or how it plays out at the strategic level. And with all due respect, if you cannot get beyond n=1, you have to question what the added value of patient participation can be”* (PAB2). For care pathway discussions, personal experience with the relevant condition is more critical, as mentioned by a network administrator: *“when you are joining a [pathology specific working group],(…) it is really about a specific cancer, and it is crucial to have someone with experience with thát cancer involved. Otherwise, you completely miss the mark.”* (NA5). The organization of patient participation also influences participant attribute requirements. In a Patient Advisory Board, members need skills like collaboration and representation, while in a panel, personal experience is more important. These influences can also flow indirectly: aim can define its organization, which in turn could shape membership requirements. However, membership attributes do not dictate the aim or structure of patient participation. Finally, the combination of aim, organization, and participant attributes determines the impact of patient participation, including its (perceived) effectiveness, legitimacy, alignment with organizational goals, and potential patient burden.

## Discussion

This study provides insights into interorganizational patient participation, highlighting implementation challenges, particularly at the strategic level. By examining how patient involvement unfolds within a network governance context, the study bridges two areas that are rarely integrated: organizational-level patient participation and interorganizational network governance. This perspective adds a layer of understanding that complements insights from single-organization approaches. Three main findings emerged. First, patient participation often begins without a clear rationale or plan, and the ability to define these depends on the domain in which participation takes place. Second, it can place an overlooked burden on patients. Third, meaningful patient participation requires alignment between goals, structures, and participant attributes.

Our findings show that patient participation in networks often starts without a clear rationale or structure, and that the ability to define clear aims depends on the domain in which patient participation is intended. Our study raises questions about this tendency to involve patients without reflecting on the aim. This may indicate that participation functions as institutionalized behavior, or even an institutionalized myth, sustained by societal norms or legitimacy rather than proven effectiveness (DiMaggio & Powell, 1983; Meyer & Rowan, 1977; Peeters et al., 2023a). This idea of interorganizational patient participation as institutionalized behavior should be further investigated in future research across other interorganizational contexts. In the studied network, participation occurred in two ways: at the operational level, focused on care optimization and alignment across organizations, and at the strategic level, addressing broader, cross-condition strategic or system-level issues. These forms differ both structurally (a single patient in a group of specialists vs. a Patient Advisory Board) and in their proximity to care. The structure often determines the degree of power granted to patient participation (Carman et al., 2013). At the operational level, a patient joined meetings with health care professionals, whereas at the strategic level, advisory board meetings were held separately from the Regional Oncology Committee. Previous literature shows that such separation can risk creating silos between the Patient Advisory Board and the network, potentially limiting genuine collaboration and shared decision-making (Tritter & McCallum, 2006). The proximity to care appears to shape the clarity of aims, with operational care-related discussions enabling more concrete aims than abstract strategic topics. The lack of clear aims and structures raises concerns about tokenism, especially given the limited evidence of meaningful outcomes (Boote et al., 2002; Van De Bovenkamp & Trappenburg, 2009). Our findings are in line with existing frameworks at the organizational level, which emphasize that clear goals, roles, and approaches are essential for meaningful participation (Elwyn et al., 2017; Westerink et al., 2023). Specifically, our findings indicate that when these elements remain unclear, patient involvement is hindered and stakeholders perceive less added value in participation. This extends the principles of existing frameworks beyond single organizations, suggesting that their emphasis on clarity should also guide interorganizational collaboration.

Second, our findings indicate that interorganizational patient participation often fails to account for the burden it places on patients, including the demands on their time and energy. Although generally seen as positive, patient participation can also have negative consequences (Westerink et al., 2023), as prior research highlights, ranging from emotional strain to unmet expectations, in addition to the significant time and energy demands (Blackburn et al., 2018; Brett et al., 2014). In the network under study, patients were asked to join a Patient Advisory Board before a clear aim or structure was defined, raising ethical concerns about asking for such investment without clarity on their role or impact. Our study shows that these potential downsides were not considered upfront, prompting the question of whether the potential burden may outweigh the benefits. This tension between benefits and burdens represents an important area for future research, particularly to explore the processes and conditions that amplify or mitigate these effects. Moreover, even when goals and roles are clearly defined, participation remains ethically questionable if patient input is not visibly used or valued (Westerink et al., 2023). Ethical and meaningful participation, therefore, requires not only a clarity regarding the aim, organization, and roles, but also a genuine commitment to act on patient contributions.

Third, meaningful patient participation requires alignment between aim, structure, and patient attributes, which together determine impact. When misaligned, patients may invest time and energy without clarity on their role or influence, increasing their burden (Brett et al., 2014). This mirrors organizational patient participation principles (Westerink et al., 2023), suggesting that interorganizational participation is not fundamentally different from participation in single organizations. Yet, research shows that reaching a clear aim is challenging in networks, as they involve navigating diverse interests of stakeholders from multiple organizations (Alexander et al., 2016). Our findings add that defining a clear aim for participation is particularly challenging at the strategic level, making impact harder to achieve. Given that hospitals participate in an average of 64 networks (van der Woerd et al., 2024) and interorganizational patient participation is increasingly emphasized (Karam et al., 2018), this raises important questions. When network aims are too distant from patients’ experiences, participation risks becoming symbolic rather than meaningful (Westerink et al., 2023). Stakeholders valued operational-level over strategic-level participation, likely because it connects more directly to experiential knowledge. These findings highlight the need for thoughtful alignment of aims, structures, and participants, while recognizing that meaningful participation at all levels may not always be feasible. This may be because the benefits at the strategic level do not outweigh the patient burden or because professionals lack expertise to identify where and how patients can contribute effectively.

This study has three main limitations. First, as a single-case study, transferability may be limited. However, we deliberately interviewed network administrators from other Dutch oncology networks. Those responses showed similar perceptions, which supports the plausibility of our findings beyond the focal case. Second, most interviewees were involved in a regional oncology networks, some even in the Patient Advisory Board, which may lead to a more favorable view of patient participation, suggesting others might face even greater challenges. Third, since patient participation at the network’s strategic level was newly introduced in this case, it provided valuable insights into different implementation perspectives in the absence of standardized approaches. However, this novelty may limit the transferability of findings to more established settings. Therefore, this study serves as a foundation for future research on patient participation in interorganizational contexts.

## Conclusion

This in-depth case study explored interorganizational patient participation, highlighting both its potential and limitations. While there is widespread support for involving patients, challenges remain in defining the aim and organizing participation in ways that lead to meaningful impact. The idea of patient participation as an institutionalized myth raises questions about whether it truly adds value or merely serves as tokenism in response to external pressures. This reflects the ‘*patient participation puzzle’*: the tension between the ideal of inclusive patient involvement and its potential burden. Patient participation should therefore only be pursued when it serves a clear purpose, is backed by a genuine intent to act upon it, and has the capacity to make an impact; otherwise, it risks becoming ineffective and unnecessarily burdensome. Future research should explore the processes and conditions that amplify or mitigate the tension between the potential benefit and the burden patient participation places on patients, as well as examine whether similar dynamics occur in other interorganizational settings.

### Practice implications

Patient participation offers many benefits and is often regarded as common sense in health care, but its implementation can be a complex puzzle with potential patient burdens and no guarantee of meaningful impact. To avoid these pitfalls, vigilance is required. Drawing on previous organizational-level literature and on our findings, we propose actions for health care networks and managers to enable meaningful interorganizational participation.

First, health care networks should prevent tokenistic participation. Because participation requires time, energy, and emotional investment, networks must clearly define its purpose, goals, organization, and required attributes (Westerink et al., 2025). Participatory design workshops can help patients and professionals jointly set goals, structures, and roles (Vargas et al., 2025). If these elements are unclear, networks should consider whether the patient burden outweighs the benefits. Second, networks must show genuine commitment to act on patient input. Literature suggests that reducing power imbalances between professionals and patients can support this process by fostering reciprocal relationships with equal expectations, recognizing patients’ assets, building on their strengths and experiential knowledge, and removing rigid boundaries between professionals and patients (Ocloo & Matthews, 2016). Allocating time and budget for participation, and appointing a dedicated coordinator can help ensure that patient input is acted upon (Westerink et al., 2025). Third, treat participation as an ongoing process with regular check-ins to align expectations and capacities, and feedback loops to show how input is used. Transparent evaluation fosters enthusiasm and mutual learning (Westerink et al., 2025). Patients’ potential vulnerability, for instance, during an active phase of their illness, underscores the need for safeguards and regular check-ins to prevent undue burden.

In addition, accreditation bodies and policymakers carry a critical responsibility in preventing interorganizational participation from becoming mere “checkbox ticking.” Mandating patient involvement at all levels without providing adequate guidance and support risks tokenism and unrealistic expectations. Instead, they should enable genuine engagement by providing clear guidelines, practical toolkits, investing training in co-creation skills, and fostering communities of practice for shared learning. In many cases, health care providers and network administrators currently lack the training, experience, or confidence for meaningful patient involvement. Equipping them with the skills to implement and manage participation effectively may yield greater value than merely mandating it.

## Acknowledgments

The authors thank the OncoZON consortium, including Anna Hospital, Catharina Hospital, Elkerliek Hospital, Laurentius Hospital, Maastricht University Medical Center+, Maastro Clinic, Máxima Medical Center, Sint Jans Gasthuis, VieCuri Medical Center, and Zuyderland Medical Center, for their support in enabling this work. R.G.F.M. van der Ven received partial salary support from the OncoZON consortium for a broader project on network research. The consortium had no involvement in the study design, collection, analyses, and interpretation of data, writing of the report, and in the decision to submit the article for publication. All other authors have no disclosures to report. Therefore, the authors declare that they have no known competing interests that could have appeared to influence the work reported in this article.

## Supplementary Material
