# Supplementary material for: The patient participation puzzle: Organizing patient participation in health care networks
Source: Health Care Manage Rev. 2026 Jul 20;51(4):295–306. doi: 10.1097/HMR.0000000000000498 (PMC13492651; doi:10.1097/HMR.0000000000000498)
Supplement: Supplementary file 1 [file hmr-51-295-s001.docx]

**Supplementary material**

**Supplementary file 1** Simplified Network Structure

**Supplementary file 2** Interview guide Patient Advisory Board

This interview guide presents an example question for each topic, followed by a description of the key points that were explored with each interview group. Because the network consisted of different actors with distinct roles, responsibilities, and perspectives, the guide differentiated between interview groups to ensure that relevant insights were captured at the appropriate level.

**NA**	Network administrator (externally)

**PAB**	Patient advisory board

**ROC**	Regional oncology committee and internal network administrators

**PF**	Nationwide cancer patient federation

**Introduction, role distribution and involvement**

How would you describe *your role* (and tasks) in relation to (the) network(s)?

**PAB/ROC/NA:** Personal interpretation; purpose/role of the network; perceived value; current structure of the network; roles and responsibilities; governance.

**PAB:** Prior understanding of the network (before joining the PAB); prior knowledge; expectations; initial impression; previous experiences; sense of involvement; participation moments; examples from care trajectory.

**ROC:** Selection process; criteria used; relevant decision-makers; previous personal experience with patient participation; earlier involvement and experiences with patient participation; level and form of participation.

**PF:** General view on regional oncology networks; advantages/concerns; expectations; current role of/in the patient federation; activities and responsibilities; examples of involvement; mission and tasks; support/advocacy; types of participation activities; experience with patient involvement at the inter-organizational level.

**NA:** Composition/structure of their own network; roles; decision-making structure; organizational structure.

**The concept of patient participation**

How would you define patient participation in your own words?

**General**: General views on patient participation; perspective on patient participation at the network level; previous experience with patient participation (any level or form); perceived benefits of involving patients in care.

**Patient participation in inter-organizational networks (in general)**

What is your perspective on patient participation in multi-hospital oncology networks? In your opinion, (how) can patient participation in such networks contribute to improving care for (cancer) patients?

**General:** Opinion on the potential impact of patient participation; opinion on the current setup of patient participation within the network (at both strategic and operational levels); eligibility criteria for inter-organizational patient participation (strategic and operational levels); necessary qualities, skills, and competencies for participation (strategic and operational levels); potential benefits, challenges, and disadvantages of network-level patient participation; practical challenges, risks, and potential downsides; description of what an ideal model of inter-organizational patient participation would look like.

**PAB/ROC/NA:** Current setup of patient participation in the network; forms/mechanisms of patient participation in the network; integration within the network; origin of patient participation in the network; internal initiative vs. external requirements; accreditation or regulatory influences; motivations and drivers; alignment with network goals (strategic- and operational-level); perceived effectiveness (strategic- and operational-level); strengths and weaknesses (strategic- and operational level); role-dependent requirements (e.g. chair, network administrator, medical specialist); additional competencies specific to the patient advisory board.

**NA:** Current status of patient participation in their network; reasons for (non) involvement; barriers or constraints; levels of involvement; operational-level patient involvement; strategic-level patient involvement; openness toward implementing patient involvement at network level; attitudes toward potential future setup.

**PF:** (Opportunities for) collaboration with other federations/organizations; opportunities for cooperation; potential forms of collaboration; added value of partnering with national federations, patient organizations or advocacy groups; recommendations; lessons learned and best practices from experience.

**Patient participation in the patient advisory board (strategic level)**

(If a patient advisory board were to be established to incorporate patient participation at the strategic level,) which themes or topics should the patient advisory board focus on? In your view, on which themes could it meaningfully influence decision‑making, and on which it could not?

**General**: Opinion on added value of the PAB; opinion on how the PAB should be structured/organized and which purpose it should serve; opinion on what rights/mandate a PAB should have; expectations of such PAB; effectiveness of the PAB; roles and tasks of the PAB; views on the ideal situation of strategic-level patient participation.

**PAB/ROC**: description of PAB in own words; PABs purpose, rights and mandate; origination of the PAB; who decided on the PAB’s structure, roles, responsibilities, purpose, and mandate; formalization of purpose, structure and tasks of the PAB; level at which the PAB provides support (operational, strategic, or other); connection/relation between PAB and ROC; connection/relation between PAB and other parts of the network; connection/relation between PAB and organizational-level patient participation (in hospitals); composition of PAB; perceptions of what is going well; areas for improvement; reflections on what could have been done differently.

**PAB**: Perceived own role in PAB; the way approached for the PAB (including information received); perceived space and opportunities to participate in the network.

**ROC**: Awareness of PAB activities; satisfaction with communication about the board’s work, discussions, decisions, and relevant developments.

**Patient participation in the tumor-specific working groups (operational level)**

What is your perspective on patient participation at the operational level (in tumor-specific working groups)?

**General**: Purpose of operational-level patient participation; perception on how operational-level patient participation (should) work in practice; differences and connections between organizational- and strategic-level participation; advantages and disadvantages of operational-level patient participation; advantages and disadvantages of operational-level patient participation.

**PAB/ROC**: Perception on operational-level patient participation as structured in the network; perception on effectiveness.

**NA**: How patient participation is structured in their own networks; perception on effectiveness.

**Organizational patient participation**

Should patient participation at the local level (organizational level) be connected to patient participation at the network (inter-organizational level)? If so, what should it look like?

**PAB/ROC**: Organizational patient participation in their own hospital; membership of local patient advisory board (in their own hospital); similarities and disparities between participation in their own hospital (organizational level) and the network (inter-organizational level); perception of own role in the connection of organizational- and inter-organizational level participation.

**NA**: Awareness of how organizational-level patient participation is arranged across the hospitals in the network; differences and connections between organizational-level and inter-organizational level patient participation in their own networks.
**PF**: Experiences with organizational-level patient participation; network‑level recommendations for patient participation based on organizational‑level experiences.

**Future plans for inter-organizational patient participation**

Do you already have ideas on how patient participation could be organized/improved in the future? How do you see the role of patient participation at the network level in 5-10 years?

**General**: Tasks, roles, rights and mandate of patient participation in the future; relation and connection of network-level patient participation with other parts of the network; organization of network-level patient participation in the future; expected value of network-level patient participation; distinction between strategic- and operational level participation; potential future challenges.

**NA**: Plans on establishing network‑wide patient participation (if not yet in place); current status.

**Closing**

Do you have any additional comments, ideas, or questions following this interview?

**Supplementary file 3** Coding tree

| **Themes** | **Subthemes** | **Categories*** |
| --- | --- | --- |
| **Aim of patient participation** | The reason for the emergence of patient participation | Patients need to be heard |
|  |  | External factors (e.g., required for accreditation or imposed by government) |
|  |  | Checking the box / window dressing |
|  |  | Reasons for not (yet) initiating patient participation |
|  | Purpose of patient participation at the network’s strategic level | Articulating patients' voices, perceptions, and/or experiences |
|  |  | Providing perspective on questions posed by the network |
|  |  | Elevating local patient advisory councils |
|  |  | Giving solicited and unsolicited advice |
|  |  | Learning from each other's local patient participation |
|  |  | Representing regional patient perspectives on a national scale |
|  |  | (Still) unclear, unknown, or non-existent |
|  | Necessity of patient participation at network level | The national patient perspective may differ from the regional patient perspective |
|  | The relationship between patient participation at the different levels (organizational, network’s operational level, network’s strategic level, and nationwide) | The role of the patient in their own organization vs. in the collective/network |
|  |  | Focus of Patient Advisory Board at the network’s strategic level, compared to the local or network’s operational levels |
|  |  | Patient participation is already embedded at the organizational level, but not yet at the network level |
| **Organization of patient participation** | How patient participation originated | Organically emerged, no agreements on goal, form, structure; that will follow once it is established |
|  |  | Patient participation must start with a clear goal in mind |
|  |  | Information gathered from each affiliated center |
|  |  | Created in a hurry |
|  | Level of patient participation | Local organizations |
|  |  | Network’s operational level |
|  |  | Network’s strategic level |
|  |  | Supra regional / national |
|  | Organization/structure of patient participation | Still very new, and therefore still discovering/no concrete agreements regarding the organization |
|  |  | Level of participation (e.g., informing, evaluating, cocreating, etc.) |
|  |  | Patient Advisory Board |
|  |  | Patient panel / surveys |
|  |  | One or a few patients join network meetings |
|  |  | Focus groups |
|  |  | Patients are (only) informed |
|  |  | Periodic meetings with the national patient federation (instead of patients from the own network). |
|  |  | Structure should be organic and flexible |
|  |  | Need for a clear and established structure. |
|  |  | The responsibility for the organization lies with … |
|  |  | There is a need for (nationwide) guidelines on how to organize patient participation in networks |
|  |  | Other |
|  | Tasks and roles of patient participation | Solicited and unsolicited advice |
|  |  | Collaborating with healthcare professionals to jointly develop strategy/policy. |
|  |  | The patients should suggest themselves what they will discuss |
|  |  | Evaluation of existing or already decided upon strategy/policy |
|  |  | Advising the Regional Oncology Committee |
|  |  | Dissemination from the Patient Advisory Board to local patient participation platforms |
|  |  | Unclear / no consensus on the role of patient participation |
|  |  | Role and tasks of the Patient Advisory Boards chair person |
|  | Mandate, authority and rights | Members of the Patient Advisory Board speak on their own behalf. |
|  |  | Members of the Patient Advisory Board speak on behalf of their own organization / their constituency |
|  |  | Members of the Patient Advisory Board speak on behalf of the entire network |
| **Participant Attributes** | Membership/composition | A representation of the population as realistically as possible |
|  |  | A small select group |
|  |  | A delegation of local patient councils (of each of the affiliated center) |
|  |  | No agreements on the composition of the Patient Advisory Board |
|  | Selection process | Approached through local patient councils |
|  |  | Difficult to achieve a heterogeneous selection (e.g., from each organization and every type of cancer) |
|  |  | Recruitment is challenging due to the vulnerability of this patient group. |
|  |  | No selection criteria in place |
|  |  | You may be critical about which patients you select. |
|  | (Required) competences, skills and characteristics of members | Members of the Patient Advisory Board do not need specific competences, skills or characteristics. |
|  |  | Still unclear which skills or qualities a member of the Patient Advisory Board should have |
|  |  | Members of the Patient Advisory Board should be/do not necessarily have to be (former) oncology patients or have had direct contact with oncology through a close relative |
|  |  | A specific professional background is useful for participation at the network’s strategic level (e.g., healthcare worker or lawyer) |
|  |  | Should be able to think in terms of the collective, not just from one's own perspective |
|  |  | Able to read complex strategy documents and follow medical conversations |
|  |  | Able to represent their (broad) constituency |
|  |  | Able to communicate |
|  |  | Intrinsic motivation / voluntary participation |
|  | Other roles in a patient advisory council | Chairperson |
|  |  | Medical specialist |
|  |  | Network administrator |
|  |  | Other |
| **Impact of network level patient participation** | Added value of patient participation at network level | Added value of patient participation at network level (compared to organizational or national level) |
|  |  | Patients are taken seriously |
|  |  | No added value/impact of patient participation |
|  | (Fear of) negative impact of patient participation | Fear among professionals that patient participation might negatively interfere with medical policies and issues such as salaries. |
|  | Patient burden | Too much responsibility is placed on the patient |
|  |  | Investment (time, energy, travel costs) without compensation |
|  |  | Feeling unheard/not taken seriously |
| **Other** | Added value of the network for the patient | The network is far removed from the patient / has little direct value for a patient |
|  |  | The network is important for quality improvement (for the patient). |
|  |  | Patients should, in principle, not notice that they are participating in a network; it should simply be well organized. |
|  | Other | Other |
| Categories are condensed from open codes. | | |
